# Automatic sleep staging using ear-EEG

**DOI:** 10.1186/s12938-017-0400-5

**Published:** 2017-09-19

**Authors:** Kaare B. Mikkelsen, David Bové Villadsen, Marit Otto, Preben Kidmose

**Affiliations:** 1 Department of Engineering, Aarhus University, Finlandsgade 22, 8200 Aarhus N, Denmark; 2 Department of Clinical Medicine, Aarhus University, Nørrebrogade 44, 8000 Aarhus C, Denmark

**Keywords:** EEG, Ear-EEG, Mobile EEG, Sleep scoring

## Abstract

**Background:**

Sleep and sleep quality assessment by means of sleep stage analysis is important for both scientific and clinical applications. Unfortunately, the presently preferred method, polysomnography (PSG), requires considerable expert assistance and significantly affects the sleep of the person under observation. A reliable, accurate and mobile alternative to the PSG would make sleep information much more readily available in a wide range of medical circumstances.

**New method:**

Using an already proven method, ear-EEG, in which electrodes are placed inside the concha and ear canal, we measure cerebral activity and automatically score the sleep into up to five stages. These results are compared to manual scoring by trained clinicians, based on a simultaneously recorded PSG.

**Results:**

The correspondence between manually scored sleep, based on the PSG, and the automatic labelling, based on ear-EEG data, was evaluated using Cohen’s kappa coefficient. Kappa values are in the range 0.5–0.8, making ear-EEG relevant for both scientific and clinical applications. Furthermore, a sleep-wake classifier with leave-one-out cross validation yielded specificity of 0.94 and sensitivity of 0.52 for the sleep stage.

**Comparison with existing method(s):**

Ear-EEG based scoring has clear advantages when compared to both the PSG and other mobile solutions, such as actigraphs. It is far more mobile, and potentially cheaper than the PSG, and the information on sleep stages is far superior to a wrist-based actigraph, or other devices based solely on body movement.

**Conclusions:**

This study shows that ear-EEG recordings carry information about sleep stages, and indicates that automatic sleep staging based on ear-EEG can classify sleep stages with a level of accuracy that makes it relevant for both scientific and clinical sleep assessment.

## Background

Sleep [1] and the quality of sleep has a decisive influence on general health [2–4], and sleep deprivation is known to have a negative impact on overall feeling of well-being, and on cognitive performance such as attention and memory [5]. However, sleep quality is difficult to measure, and the current gold standard, polysomnography (PSG) [6] requires expert assistance and expensive equipment. Moreover, characterizing sleep by means of conventional PSG equipment will inevitably have a negative impact on the sleep, and thereby bias the sleep quality assessment. Because of the need for professional assistance in PSG acquisition, and because of the laborious process to evaluate PSG data, sleep assessment is in most cases limited to a single or a few nights of sleep.

Due to these circumstances, there is an ongoing effort to explore other options for high-quality sleep monitoring [7, 8]. A very promising candidate in this field is ear-EEG [9], due to its potential portability and the fact that it conveys much of the same information as the PSG, namely EEG data [10]. It is likely that the ear-EEG technology will have a much lower impact on the quality of sleep, giving a more accurate picture of the sleep, and also be suitable for sleep assessment over longer periods of time. Recently, the feasibility of ear-EEG for sleep assessment has been studied in a few exploratory papers [11–13], all indicating that ear-EEG is a very promising candidate.

This paper is based on a new dataset comprising nine healthy subjects recorded simultaneously with both PSG and ear-EEG for one night. This is significantly more sleep data than in previous studies. Trained clinicians manually scored the sleep following the guidelines of the American Academy of Sleep Science (AASM) [14]. The sleep staging based on ear-EEG was based on an automatic sleep staging approach, where a statistical classifier was trained based on the labels from the manual scoring (for other examples of this, see [15–17]). The automatic sleep staging was chosen for two reasons: (i) there was not any established methodology for sleep staging based on ear-EEG, while the machine learning approach provided rigorous and unbiased sleep staging. (ii) The question of whether a given method can also be used without manual scoring is important whenever wearable devices for long term monitoring are discussed.

In the “Results” section below, additional support for this reasoning is presented, based on waveforms.

## Methods

### Research subjects

For this study, nine healthy subjects were recruited, aged 26–44, of which three were female. Measurements were all conducted in the same way: subjects first had a partial PSG [consisting of six channel EEG, electrooculography (EOG) and electromyography (EMG) on the chin] mounted by a professional at a local sleep clinic. Subsequently the subject was transported to our laboratory where the ear-EEG was mounted.

The subjects went home and slept with the equipment (both PSG and ear-EEG) for the night, and removed it themselves in the morning. The subjects were instructed to keep a cursory diary of the night, detailing comfort and whether the ear-EEG ear plugs stayed in during the night.

### EEG hardware

**Fig. 1:**
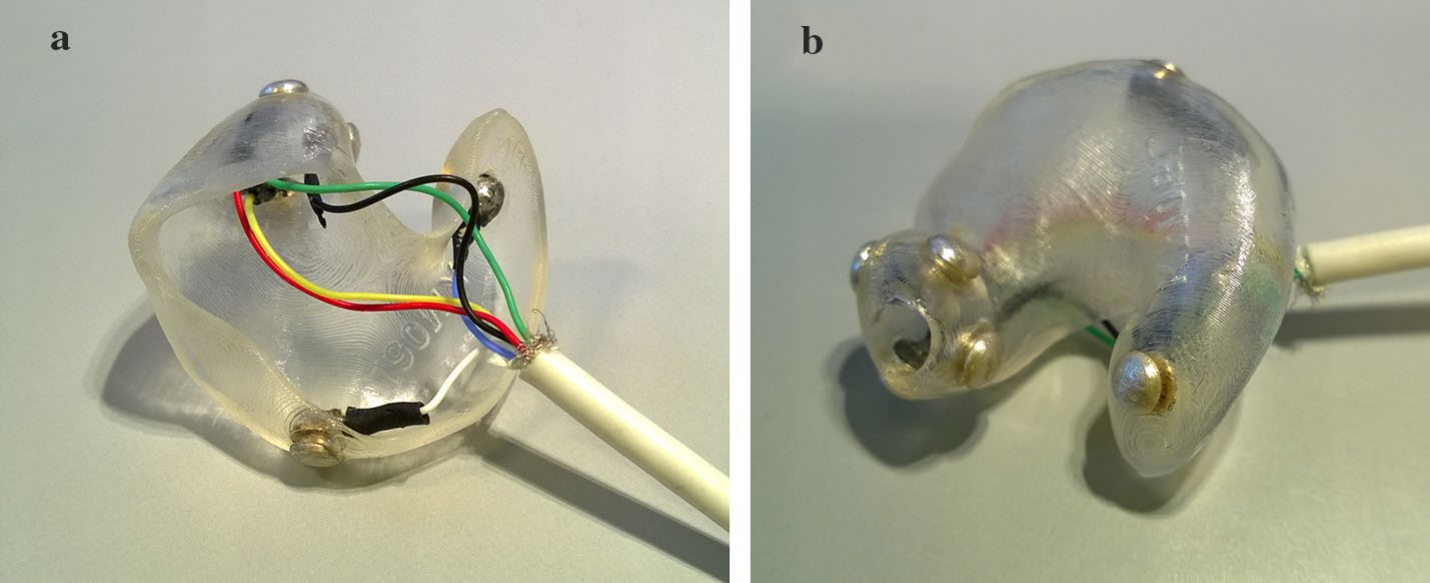
Example left-ear ear plug with silver electrodes.

The ear plugs used in this study were shaped very similarly to those used in [18], with the difference that the plugs here were made from soft silicone, and the electrodes were solid silver buttons soldered to copper wires. See Fig. 1 for an example of a left-ear plug. Before insertion, the outer ears were cleaned using skin preparation gel (NuPrep, Weaver and Company, USA) and electrode gel (Ten20, Weaver and Company, USA) was applied to the electrodes. Ear-EEG electrodes were ELA, ELB, ELE, ELI, ELG, ELK, ERA, ERB, ERE, ERI, ERG, ERK, as defined in [19].

As described in [18], ear-EEG electrodes were validated by measuring the auditory steady state responses (ASSR) using 40 Hz amplitude modulated white noise, which was performed while the subject was still in the laboratory. All electrodes (including ear-EEG) were connected to the same amplifier (Natus xltek, Natus Medical Incorporated, USA), and ear-EEG electrodes were Cz-referenced during the recording. The PSG consisted of two EOG electrodes, two chin EMG electrodes and 8 scalp electrodes (O1, O2, C3, C4, A1, A2, F3, F4 by the 10–20 naming convention). The data was sampled at 200 Hz.

### Sleep scoring

#### Manual scoring

All PSG-measurements were scored by trained experts at the local sleep clinic, according to the AASM guidelines [14]. The scorers did not use the ear-EEG data in any way, and did not receive any special instructions regarding this data. Scoring was done based on 30-s non-overlapping epochs, such that each epoch was assigned a label from the set: W, REM, NREM1, NREM2, NREM3. We direct the reader to the established sleep literature (such as [14]) for a discussion of these labels.

#### Automatic scoring

To investigate the hypothesis that ear-EEG data can be used for sleep scoring, machine learning was used to train an automatic classifier to mimic the scoring of the sleep experts. The analysis pipe line used for this is described below.

### Channel rejection

Even though the ear-EEG electrodes were qualified in the lab by measuring an ASSR, it was found in the analysis of the sleep EEG, that some of the ear-EEG channels were noisy. This was probably due to a deterioration in the electrode-body contact from the time when the subject left the lab until they went to bed. The deterioration may also be related to deformation of the ear, when the subject laid their head on the pillow. Because of this deterioration, it was necessary to perform a channel rejection prior to the analysis of the data. This was done in the following way:

All intra-ear derivations were calculated, and the power in the 10–35 Hz frequency band was calculated. If $p_{ij}$ is the power calculated for the derivation consisting of channels *i* and *j*, let $m_i=\text {median} \left( \{p_{ij} \}_{j}\right)$. Electrode *i* was then rejected if $m_i>5\cdot 10^{-12}\,\text {V}^2/\text {Hz}$. This uses the fact that a high-impedance electrode will tend to have much more high-frequency noise, and that this will be the case for all derivations that it takes part in. Elegantly, it does not require a simultaneous ’ground truth’ electrode, such as a scalp measurement, to determine good and bad electrodes. The value of $5\cdot 10^{-12}\,\text {V}^2/\text {Hz}$ was determined by observing which value cleanly separated the electrodes into two groups, commensurate with the knowledge from the ASSR measurements and the subject diaries (for instance, one subject reported having removed one ear plug entirely before falling asleep). See Appendix A for a visualization of this separation. In total, 14 electrodes were rejected out of a possible 72, resulting in a rejection rate of 19%.

We note that the band-pass filtering of 10–35 Hz was only chosen and performed for the sake of this channel rejection. The non-filtered data set was passed to the next stage of the analysis, as described below.

### Feature extraction

The eight ear-EEG channels were distilled into three derivations ($\left\langle \cdot \right\rangle$ denotes average):

$$\begin{aligned} \text {L-R: }&\left\langle ELA, ELB, ELE, ELI, ELG, ELK \right\rangle \\&{\qquad \qquad \qquad } -\left\langle ERA, ERB, ERE, ERI, ERG, ERK \right\rangle \\ \text {L: }&\left\langle ELA, ELB \right\rangle -\left\langle ELE, ELI, ELG, ELK \right\rangle \\ \text {R: }&\left\langle ERA, ERB \right\rangle -\left\langle ERE, ERI, ERG, ERK \right\rangle \end{aligned}$$

Note that the L and R-channels describe the potential differences between concha and channel electrodes in each ear. If an electrode was marked as bad, it was excluded from the averages. If this meant that one of the derivations could not be calculated (for instance, if both ELA and ELB were missing), that derivation was substituted with a copy of one of the others. This was only done in the case of subject 5, which was missing data from the right ear plug.

When choosing features, we were inspired by [15], and chose the list of features shown in Table 1. Of these, a subset were not used by [15] and are described in Appendix B. In general, the time and frequency domain features were based on a 2–32 Hz-bandpass filtered signal, while the passbands for EOG and EMG features were 0.5–30 and 32–80 Hz, respectively. A 50 Hz notch filter was also applied. All frequency domain features were based on power spectrum estimates using Welch’s algorithm with segment length 2 s, 1 s overlap and applying a Hanning window on each window.

It is important to stress that the EOG and EMG proxy features discussed in this paper were extracted entirely from ear-EEG data—no EOG or EMG electrodes were used in the analysis. This was to distill as much information about EOG and EMG variation as possible from the ear-EEG data.

**Table 1:** Features used in this study

Label	Short description	Type
F1	Signal skewness	EEG time domain
F2	Signal kurtosis	
F3	Zero crossing rate	
F4	Hjorth mobility	
F5	Hjorth complexity	
F6	75th percentile	
F7	Channel correlation	
F8	EMG power	EMG proxy
F9	Minimal EMG power	
F10	Relative EMG burst amplitude	
F11	Slow eye movement power	EOG proxy
F12	Rapid eye movement power	
F13, F14, F15, F16	Relative power in $\alpha ,\beta ,\theta ,\delta$-bands	EEG frequency domain
F17, F18, F19, F20, F21, F22	Power-ratios: $\alpha /\delta ,\delta /\beta ,\delta /\theta ,\theta /\alpha ,\theta /\beta ,\alpha /\beta$	
F23	$(\theta +\delta )/(\alpha +\beta )$	
F24	Spectral edge frequency	
F25	Median power frequency	
F26	Mean spectral edge frequency difference	
F27	Peak power frequency	
F28	Spectral entropy	
F29	Spindle probability	Sleep event proxies
F30	Frequency stationarity	
F31	Lowest adj. frequency similarity	
F32	Largest CWT value	
F33	Longest sleep spindle	

F1-6 and F13-25, 27, 28 are copied from [15], see Appendices A, B for a precise mapping between these features and those in [15]

All 33 features were calculated for each of the three derivations. As described in Appendix B, an attempt was made to reduce the number of features. However, this did not yield satisfactory results, and instead all 99 features were used in the study.

### Classifier training

#### Type of classifier

We used ensembles of decision trees, called a ‘random forest’ [20], with each ensemble consisting of 100 trees. The implementation was that of the ‘fitensemble1’ function in Matlab 2015b, using the ‘Bag’ algorithm. This means that each decision tree is trained on a resampling of the original training set with the same number of elements (but with duplicates allowed), and each tree has a minimum leaf size of 1. For each tree, splitting is done such that the Gini coefficient [21] is optimized, and continues until all leaves (subgroups) are either homogeneous or have the minimum leaf size.

#### Cross validation

**Fig. 2:**
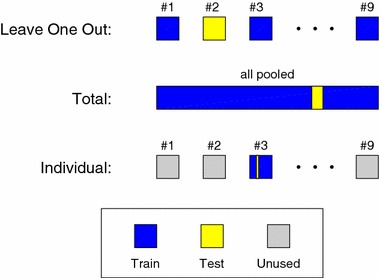
Graphical illustration of the three cross validation methods. The ‘Leave-one-out’ method use all epochs from eight subjects for training, and all epochs from the remaining subject for testing. This is done for all nine subjects (ninefold cross validation). In the ‘Total’ method, all epochs from all subjects are pooled together and partitioned into 20 subsets. The classifier is trained on 19 subsets and tested on one subset. This is done for all 20 combinations of subsets (20-fold cross validation). In the ‘Individual’ method the classifier is subject specific. For each subject, the epochs are partitioned into ten subsets, the classifier is trained on nine subsets and tested on one subset. Thereby the algorithm is validated ten times on each of the nine subjects (90-fold cross validation)

We explored three different ways to select test and training data for the classifier (described graphically in Fig. 2):

- *Leave-one-out* Data was partitioned into nine subsets, each subset corresponding to a single subject. Thus the classifier had not seen any data from the person which it was tested on.
- *Total* All epochs from all subjects were pooled, and partitioned into 20 subsets. A classifier was trained based on 19 sub-sets, and tested on the last subset. Cross-validation was performed over all 20 combinations.
- *Individual* Same as ‘Total’, but only done on data from a single subject, which was split into ten subsets. Thus, there were 90 different test sets.

The three validation schemes each provide a different perspective on the sleep staging performance and the applicability of the method.

‘Individual’ is thought as a model of the scenario in which users have personal models/classifiers created. This builds on an assumption that measurements from one night will have similar characteristics to those from a different night. This seems like a reasonable assumption, given the literature [22–24]. As shown in Fig. 2, test and training data was only picked from the same subject. Of course, as part of the calculation of the population Cohen’s kappa value, all data was eventually used as test data (each test having its own training data).

In ‘Leave-one-out’, a pre-trained classifier was applied to data from a new subject, which is probably the most relevant scenario. However, in this study we only had nine subjects, which is likely much too low for any given subject to be well represented by the remainder of the population.

Therefore, we have included ‘Total’, which represents the scenario where the pool of subjects is very large, in which case all normal sleep phenotypes are assumed represented in the training data. In the limit of a very large subject group, it is expected that ‘Leave-one-out’ and ‘Total’ would converge, to a result in-between the results reported here. However, to achieve this would likely require a substantial number of subjects.

During the analysis, we found that it is very important in ‘Total’ and ‘Individual’ that the test sets each form contiguous subsets of the data. If instead of the above, each subset was selected at random, it would mean that most likely each test epoch would have neighboring training epochs on both sides. This in turn would give the classifier access to the correct label for epochs extremely similar to the test epoch, preventing proper generalization, and leading to over fitting. We will return briefly to the discussion of this ‘neighbor effect’ later in the paper.

To evaluate the agreement between the expert labels and the output of the classifiers, Cohens kappa coefficient [25] was calculated for each of the three cross-validation methods.

## Results

### Measurements

All nine subjects managed to fall asleep wearing the PSG and ear-EEG equipment. One subject (number 5) reported having removed the right ear plug before falling asleep. When asked to judge their quality of sleep between the categories: unchanged–worse–much worse, 1 subject reported “unchanged”, 5 reported “worse” and 3 felt they slept much worse than usual. The subjects were not asked to describe whether their discomfort was caused by the ear-EEG device, the PSG, or both. The subjects slept (or attempted to sleep) between 2.4 and 9.6 h with the equipment on, an average of 6.9. This means that in total, 61.8 h of sleep were recorded and scored by the sleep scorer, resulting in 7411 30-s epochs. In Table 2 are shown the number of useable electrodes and scored epochs for each subject.

In the analysis below, the one-eared subject was not removed, instead all three derivations were identical for that subject.

### A first comparison

**Fig. 3:**
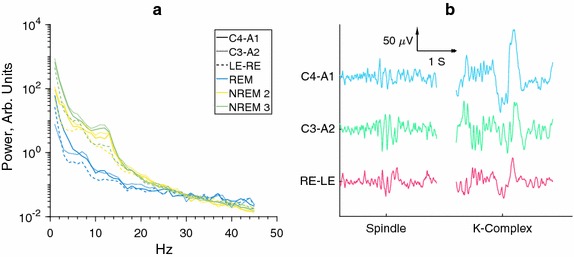
Comparisons of conventional EEG and ear-EEG. It is observed that there are clear spectral similarities between the NREM stages for scalp and ear electrodes, and that the spectral distributions are clearly distinctive from the spectral distributions of the REM stages. However, is also seen that while much of the sleep information is inherited by the ear-EEG, the stage signatures are not completely identical, leading to our use of automatic classifiers. **a** Spectral power distribution in the three major sleep stages. Normalized to have similar power for top half of spectrum. **b** Sleep events shown for different electrode pairs, simultaneously recorded. Data has been subjected to a [1; 99] Hz band-pass filter, as well as a 50 Hz notch filter

Figure 3 shows characteristics of conventional EEG and ear-EEG, during sleep. Figure 3a shows power spectra for REM, NREM2 and NREM3 for two scalp derivatives and a left-right ear-EEG derivative. A large degree of similarity is observed for the scalp and ear derivatives, in particular REM and NREM spectra are clearly separated for all three derivatives. Figure 3b shows characteristic sleep events (sleep spindle and K-complex) for the same two scalp derivatives and the left-right ear-EEG derivative. Clear similarities in the waveforms are observed across all three derivatives.

However, despite these similarities it cannot in general be assumed that sleep stage signatures will be exactly equal in conventional EEG and ear-EEG. Further, it should be stressed that not all sleep events are as clearly visible in ear-EEG as those shown. As was mentioned in the introduction, this is part of the reason why a machine learning approach is suitable for this study. More precisely, while we deem it likely that sleep experts could be trained, with some level of success, to score sleep based on ear-EEG data, it would likely require a significant amount of retraining, not suitable for this study.

### Classification results

**Fig. 4:**
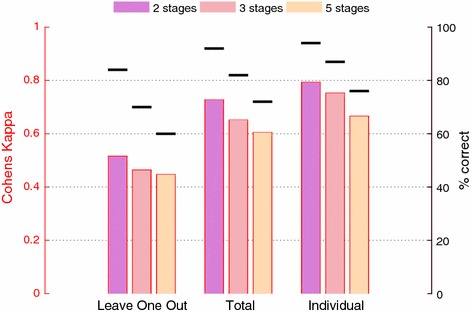
Cohens Kappa ($\kappa$) for different numbers of stages and different ways to cross validate. For comparison, the percentage of correctly labeled stages is also shown. Not surprisingly, we see that $\kappa$ increases when the number of stages decrease, and when the classifier has more prior information about the subject(s). In all cases, for calculation of both Kappa and accuracy, all epochs have been pooled together before calculation, equivalent to taking a population average weighted by number of subject epochs

Figure 4 shows kappa values ($\kappa$) for the three modes of cross validation and for 5, 3 and 2-stage classification (the stages in the last two being W-REM-NREM and W-Sleep, respectively). Results for 3 and 2-stage classification were simply obtained by relabelling the 5-stage results, so the classifiers were not retrained. Regarding the percentagewise agreement, it is noteworthy that manual scorers have been shown to have an average agreement of $82.6\%$ [26], while actigraphs using 2 stages have an agreement rate of 83.9–96.5% with PSG’s [6].

For comparison, our classifier performs somewhat worse than the ones presented in [15] ($\kappa \approx 0.85$) and [16] (correlation coefficient $\approx 0.84$), though their studies did use scalp electrodes instead of ear-EEG.

When comparing the numbers shown in Fig. 4 to those found elsewhere in other studies, it is valuable to keep in mind that the ‘neighbour effect’ stemming from scattered test data, as was discussed above (see “Cross validation” section), may not always be accounted for in the literature. In our case, using scattered test data increased the percentagewise agreement between manual and automatic labels by an average 6% points across ‘Total’ and ‘Individual’.

**Fig. 5:**
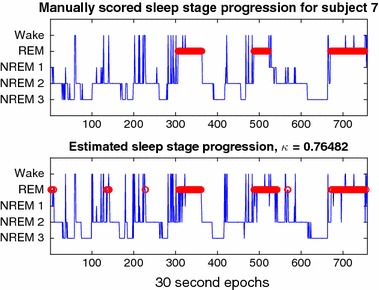
Comparison of sleep staging results from manual (top) and automatic (bottom) staging, for one subject, using a classifier only trained on data from the same subject. REM stages have been highlighted in red, as per usual convention

Figure 5 shows sleep staging traces for subject 7, using the ‘Individual’ cross-validation method. We see that generally the transitions between stable stages are accurately predicted.

**Fig. 6:**
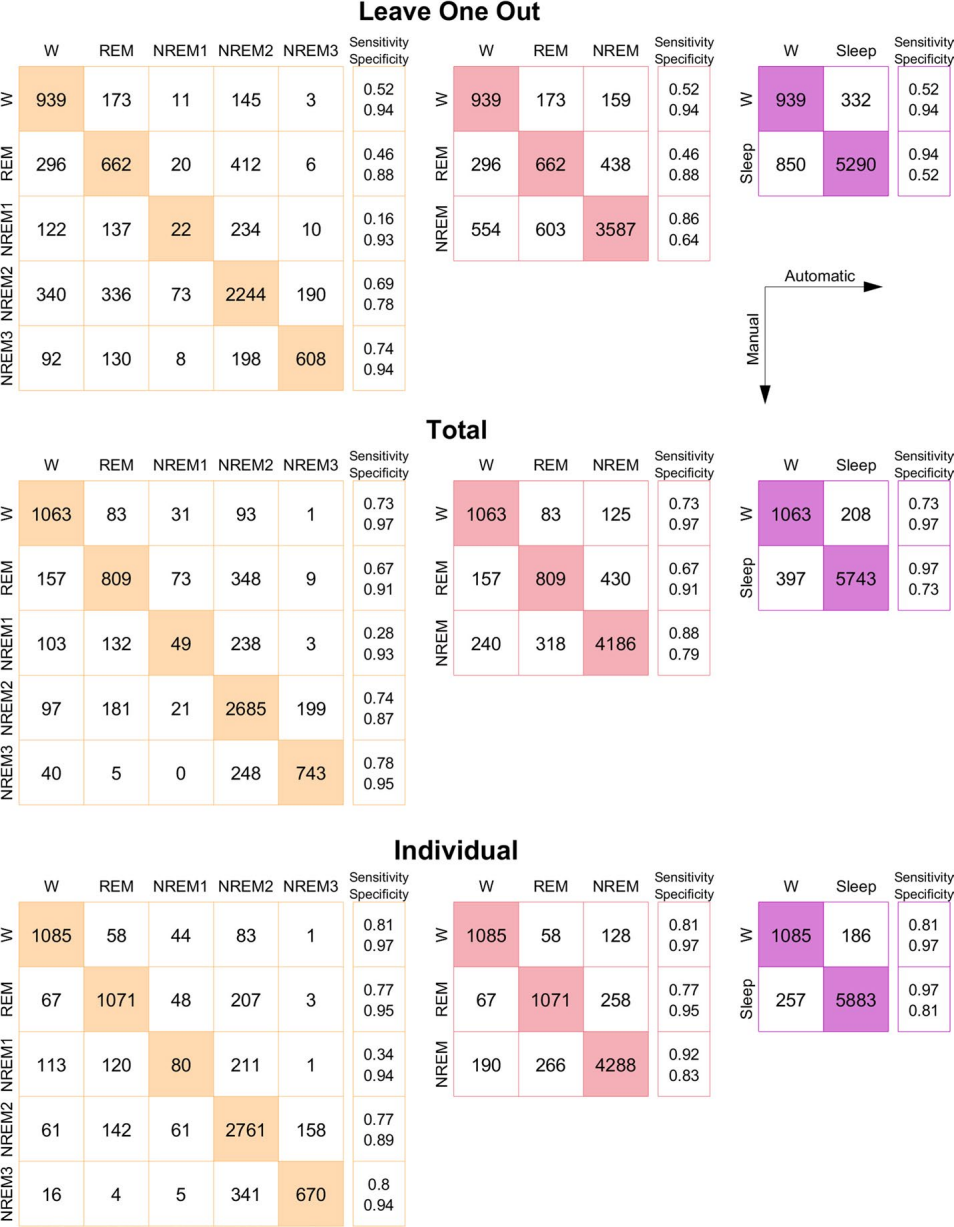
Confusion matrices for the three types of cross validation, and three model complexities. Colors match those of Fig. 4. For each matrix is given an extra column of sensitivities and specificities. We direct the reader to Fig. 4, right axis, for average accuracies. As indicated by the legend, rows correspond to manual labels, columns to automatically generated labels

Figure 6 shows the confusion matrices for the three cross validation schemes. The most difficult state to identify is NREM 1, likely stemming from the fact that there are very few examples of this (only $7\%$ of epochs). However, NREM 2 and NREM 3 are identified very well, even for ‘Leave-one-out’ cross validation.

In Table 2 is shown the $\kappa$-values for all subjects, for each method of cross validation. It is interesting to note that subject 5 was not always the worst performing subject, despite the fact that only data from one ear piece was available from this subject.

**Table 2:** $\kappa$ values for each subject, for all methods of cross-validation

Subject	1	2	3	4	5	6	7	8	9	Avr.
Useable electrodes	10	10	11	12	6	12	12	11	10	10.4
Scored epochs	1040	964	932	1150	491	926	758	293	857	823
Leave-one-out, $\kappa$	0.05	0.36	0.57	0.60	0.03	0.59	0.75	0.65	0.44	0.45
Total, $\kappa$	0.50	0.49	0.63	0.65	0.57	0.64	0.78	0.65	0.70	0.62
Individual, $\kappa$	0.52	0.52	0.67	0.72	0.55	0.65	0.76	0.70	0.76	0.65

Averages were calculated as the average of all nine columns, not by weighting each subject by number of epochs

## Discussion

We have seen that ear-EEG as a platform for automatic sleep staging has definite merit, especially if problems related to inter-subject variability can be addressed. Compared with other studies [15, 27, 28], the subject cohort in this study is rather small at only nine individuals. However by resampling the cohort, it is possible to estimate the classifier performance for larger cohorts; following the procedure outlined in [29] we find that a cohort size of 30 would likely have increased the 5-stage ‘Leave-one-out’-$\kappa$ to 0.5.

An intriguing question which was not addressed here is intra-subject variability. In other words, how well does a classifier trained on data from Tuesday perform on data from Wednesday? It seems safe to say that it will at the very least be comparable to the ‘Leave-one-out’-scheme described here, but possibly much closer to the ‘Individual’ scheme. Based on studies concerning individual differences in physiological measures during sleep [22–24], it seems likely that intra-subject variability will be low. In this scenario, one could imagine uses where a single night (possibly just a day-time nap) with both PSG and ear-EEG could be used to calibrate a classifier to each individual user. One example could be a clinical setting where the usual one night of PSG could be supplemented with a longer ear-EEG study spanning several weeks or more.

All data in this study was obtained from healthy individuals, and thus the study does not provide any information as to how ear-EEG would perform in the presence of pathology. However, given the demonstrated ability of ear-EEG to reliably classify sleep staging, it is likely that a specialist could utilize the technology to detect abnormal sleep.

A surprising issue during the study was that of user comfort. As soon as user discomfort was reported, a parallel investigation was initiated into possible remedies. These will be applied in a future study, where we expect the level of discomfort to be substantially reduced.

An additional benefit of the ear-EEG platform is the ease with which the electrodes remain attached to the skin. Whereas conventional electrodes need adhesives and/or mechanical support to ensure a reliable contact, ear-EEG benefits from the precise fit of the ear piece within the outer ear, largely retaining the connection through geometry alone.

## Conclusions

The study makes the valuable contribution of having more participants than previous ear-EEG sleep studies, as well as being the first study to make a quantitative comparison to simultaneously recorded PSG.

Through the machine learning approach, the study amply demonstrates that ear-EEG contains sleep-relevant data, in line with previously published studies. However, the need for a comfortable sleep-monitoring solution is also highlighted. We are convinced, based on developments taking place after this study was conducted, that the comfort problems discussed here will be solved in future studies.

In summary, we consider the findings of this study very positive regarding the continued development of ear-EEG as a mobile sleep staging platform.

Sleep monitoring with ear-EEG will be particularly interesting in cases where it is relevant to monitor sleep over extended periods of time. In such cases automatic sleep staging turns out to be even more important and is probably a necessity. The findings in this study are also very positive in this regard.

In future studies, it would be interesting to add additional ways to compare measurements, for instance one in which the training and test sets were matched according to age and gender. This would most likely require a substantially larger pool of subjects.

## Figures and Tables

**Fig. 7:**
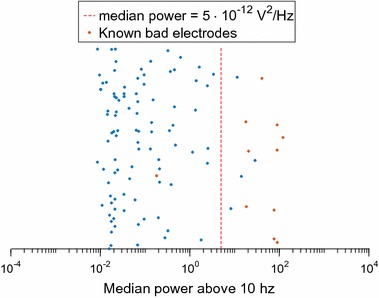
The justification for the chosen threshold for electrode rejection. Each marker corresponds to an electrode, red markers show electrodes which, based on either ASSR measurements or visual inspection, were deemed unsuitable. We see that rejecting all electrodes above this limit corresponds quite well to the initial, more loosely defined criteria

**Fig. 8:**
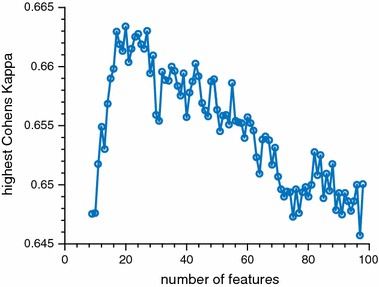
Results from feature rejection. Features were gradually removed from the pool, in order to increase $\kappa$. At each step, the highest achieved value was recorded. Note the overall very low variation in $\kappa$

## Appendix A: Channel rejection

To further elaborate on the choice of electrode rejection criteria, Fig. 7 shows $m_i$ for all channels. In the plot, electrodes that were expected to be bad either due to lost connection during the recording (an ear plug removed, for instance) or due to poor results in the initial ASSR test, have been marked in red. We see that the simple rejection criteria employed finds almost all these electrodes, and we consider this method both a more reproducible, as well as more scientifically sound approach.

## Appendix B: Additional feature discussion

### Feature elimination

Feature elimination was attempted, by systematically removing one feature, and evaluating classifier performance, looping over all features. After each loop, the feature whose absence was the least detrimental to classifier performance was removed for the remainder of the analysis. In this way, the pool of features was gradually shrunk [30].

The data material in this test was all subjects pooled (called ‘Total’ above), and classifier performance was evaluated by partitioning the data pool into 20 equal parts, and iteratively training a classifier using 95% of the data as training data and the remaining 5% as test data. Finally, Cohen’s $\kappa$ was calculated based on the combined classifier results.

Figure 8 shows the maximal $\kappa$ as a function of number of features. We see a general trend that best performance is achieved somewhere between 20 and 60 features. However, after further analysis, we have discovered that the precise set and number of features depends intimately not only on which subjects are included in the pool, but also how that pool is partitioned into 20 chunks. In other words, either the same, somewhat arbitrary choice of features is used, based on one chosen partitioning, in which case there is a risk of introducing a bias in the classifier (whichever representative subset of the data is chosen for selecting features, that subset may be overfitted when it is later used as test data), or there will be no clear indication of which set of features others should use. In the latter case, we would still have to present 99 features to our readers, and would have achieved no improvement in either classifier performance or readability. In future studies, we aim to have sufficient data to set aside a dedicated validation data set for determining feature selection, without the risk of overfitting. For now, we have chosen not to perform feature selection, but instead keep all 99 features.

### Feature details

Below is given a detailed description of those features used which were not included in [15].

- *F7: Correlation coefficient between channels* The only feature requiring multiple channels in its definition. Since there are three EEG derivations, this feature was simply calculated as a the correlation coefficient between the i’th and (i+1)’th derivations, making sure that all pairs of derivations are evaluated.
- *F8: EMG power* Total power in the [32, 80] Hz band.
- *F9: Minimal EMG power* Each epoch was split into ten segments. EMG power was calculated for each segment, and the lowest of these ten values was recorded.
- *F10: Relative EMG burst amplitude* Maximum EMG signal amplitude divided by F9.
- *F11: Slow eye movement power* Power in the frequency band [0.5, 2] relative to full power in the [0.5, 30] Hz-band. Inspired by Zhang et al. [31].
- *F12: Rapid eye movement power* Power in the frequency band [2, 5] relative to full power in the [0.5, 30] Hz-band. Inspired by Zhang et al. [31].
- *F26: Mean spectral edge frequency difference* Taken from [32].
- *F29: Spindle probability* Letting $P(x-y)$ be the set of power estimates for frequencies in the x to y Hz band, this feature is calculated as $\max (P(11-16))/(\left\langle P(4-10)\right\rangle + \left\langle P(20-32) \right\rangle)$, and is inspired by Huupponen et al. [33] (“sigma index”).
- *F30: Frequency stationarity* For each epoch, the Welch algorithm calculates power spectra for 31 segments. F30 calculates the average Pearson correlation between these 31 spectra.
- *F31: Lowest adj. frequency similarity* Using the same correlations as in F30, F31 is the lowest recorded correlation between neighboring segments.
- *F32: Largest CWT value* A continuous Wavelet Transform of the filtered EEG-signal is computed, using a complex frequency B-spline as wavelet. The wavelet has a support of 0.5 s. Inspired by Lajnef et al. [34].
- *F33: Longest sleep spindle* The signal was bandpass filtered to a band of 11–16 Hz, and the Teager energy operator (TEO) was applied to it (see [35]). At the same time, a short term Fourier transform (STFT) was applied to the unfiltered signal, and the power in the 12–14 Hz band relative to average power in 4–32 Hz band was computed (excluding 12–14 Hz). Finally, signal segments in which F32 > 15, TEO > 0.5 and STFT power > 0.3 were assumed to be sleep spindles. The maximal length of observed spindles constituted F33. This was inspired by Duman et al. [35].

As to the remaining features, the exact mapping is: (letting KN be the N’th feature from [15]): F1:K3, F2:K4, F3: K5, F4: K7, F5: K8, F6:K9, F13:K14, F14:K15, F15:K16, F16:K17, F17:K18, F18:K19, F19:K20, F20:K21, F21:K22, F22:K23, F23:K24, F24:K25, F25:K27, F27:K26, F28: K28.
